# The 42 problem in AI-assisted research

**DOI:** 10.7554/eLife.113137

**Published:** 2026-10-14

**Authors:** Giovanni Spitale, Federico Germani

**Affiliations:** 1 ITE Lab, Institute of Biomedical Ethics and History of Medicine, University of Zurich Zurich Switzerland; 2 Institute for Data Science and Artificial Intelligence, Boğaziçi University Istanbul Turkiye

**Keywords:** AI, large language models, citations, warrant, AI systems, None

## Abstract

Large language models are very good at providing concise answers. But if we only see the answer without knowing the original question or the context behind it, that answer may not be very useful. The important issue is not simply how much information an AI summary removes but whether we can still recover the missing context. Thus, AI literature tools should also report how much information was removed and how recoverable their omissions are.

Large language models are here to stay – at least for the foreseeable future. At the time of writing, multiple large language model families exist, used by millions to seek input on a myriad of tasks, issues, and problems, from translation, writing assistance, or content creation to more complex matters such as healthcare, finance, coding, or relationship advice.

Modern AI systems are powerful tools that can enable researchers to do better, faster research, with developers promising superhuman search and synthesis of the literature (Skarlinski et al., 2024). But how reliable are these tools, especially when applied to research articles designed for human communication and not machine reading (Inside eLife, 2026)?

A study comparing 4,900 machine-generated summaries of scientific papers with their originals found that most models broadened the conclusions, with three of them overgeneralising in 26–73% of their summaries. Compared with expert summaries of the same clinical articles, machine-generated versions were nearly five times more likely to contain broad generalisations (odds ratio 4.85) (Peters and Chin-Yee, 2025).

While AI systems may be able to search and synthesise large quantities of literature more quickly than humans, they may also lose context or risk overgeneralising facts. It is the gap between these realities that warrants exploration, as illustrated by an old joke. In Douglas Adams’s “The Hitchhiker’s Guide to the Galaxy”, a supercomputer spends seven and a half million years computing the “Answer to the Ultimate Question of Life, the Universe and Everything”. The answer is 42. In the end, nobody remembers the question. An answer cut loose from its question and context is inert: you cannot use it or check it.

Machine-generated summaries can remove the conditions that give a finding its meaning, and they do so unevenly across tasks, with context sometimes getting lost (Peters and Chin-Yee, 2025; Van Veen et al., 2024; Aigner et al., 2026). But what this means for how we should use and evaluate AI summaries has largely been left unexamined. We thus offer three propositions. First, the value of a synthesis does not simply increase as it becomes shorter. Second, at what point further compression of a synthesis becomes less useful is subjective to the reader (and the question they are trying to answer). Hence, no single, standard level of compression will work for everyone or every purpose, which may explain why existing benchmarks fail to capture the problem. Third, the problem is not necessarily a badly compressed summary; this can often be caught by review. It is a well-compressed summary applied to one purpose that is later used to answer a different question it was never meant to be compressed for – and for which the omitted information matters.

The 42 problem comes to mind whenever we, the authors, hear that language models will free scientists from reading. Scientific knowledge has been growing at roughly four percent a year, doubling every 17 years (Bornmann et al., 2021). A system that synthesises on demand seems to offer what scholarship has always been short of: human attention. One of us argued before the rise of large language models that search and text mining were a coping strategy rather than a solution to the problem of information overload (Spitale, 2020). The tools have changed; the argument has not.

Language models are extraordinarily good at one part of the task: producing an answer. But an answer is only useful if it addresses the right questions, is understood in the right context and leads to insight in the person reading it. This is where language models fail. The bottleneck, i.e., the process that limits how much useful work can get through, does not disappear. It merely shifts from finding the right information to posing the right question, and from retrieving knowledge to the capacity and enough attention to read, evaluate and integrate it. The human reader remains the narrowest part of the pipe.

The economics of AI look favourable at first. If the value of a synthesis is the insight it provides divided by the effort required to read it, then making a synthesis faster to consume should increase its value. Within a certain range, that is exactly what they are designed to achieve. But then 42 returns.

Compression works by leaving things out. Up to a point, this is pure efficiency. Past that point, the compressed answer loses the context connected to its question and the conditions under which it is valid. The overgeneralisation found in the study by Peters et al. is this loss made visible (2025). As context disappears, findings can be overstated – not because the model makes errors, but because it removes the qualifications that define the scope of the original finding. The result may read fluently, cost almost nothing, but it is almost worth nothing. You are back at 42: a confident answer, with no question.

It helps to visualise this (Figure 1A). Let L be the length of a synthesis. Three quantities move with the length of a synthesis (L). Recovery, R(L) stands for how much of the source material the reader can reconstruct; it rises steeply and then saturates. Cost, C(L), describes the attention the reader pays, and it rises without saturating. Usability, U(L), is whether the text can be acted on at all, and it does not decline gracefully: while the text carries the conditions of validity, it remains near 1, then falls away once L drops below a threshold, L*. A claim stripped of its scope conditions is not a weaker claim; it is a different one. The value of a synthesis is then V(L)=R(L)·U(L)/C(L). Above L*, value is governed by R over C, and the case for compression is exactly as good as its advocates say. At L*, U collapses and takes V with it. This is the ‘cliff’, the limit of useful compression, and it is why more concise is not a direction of improvement but one with an end.

**Figure 1.**
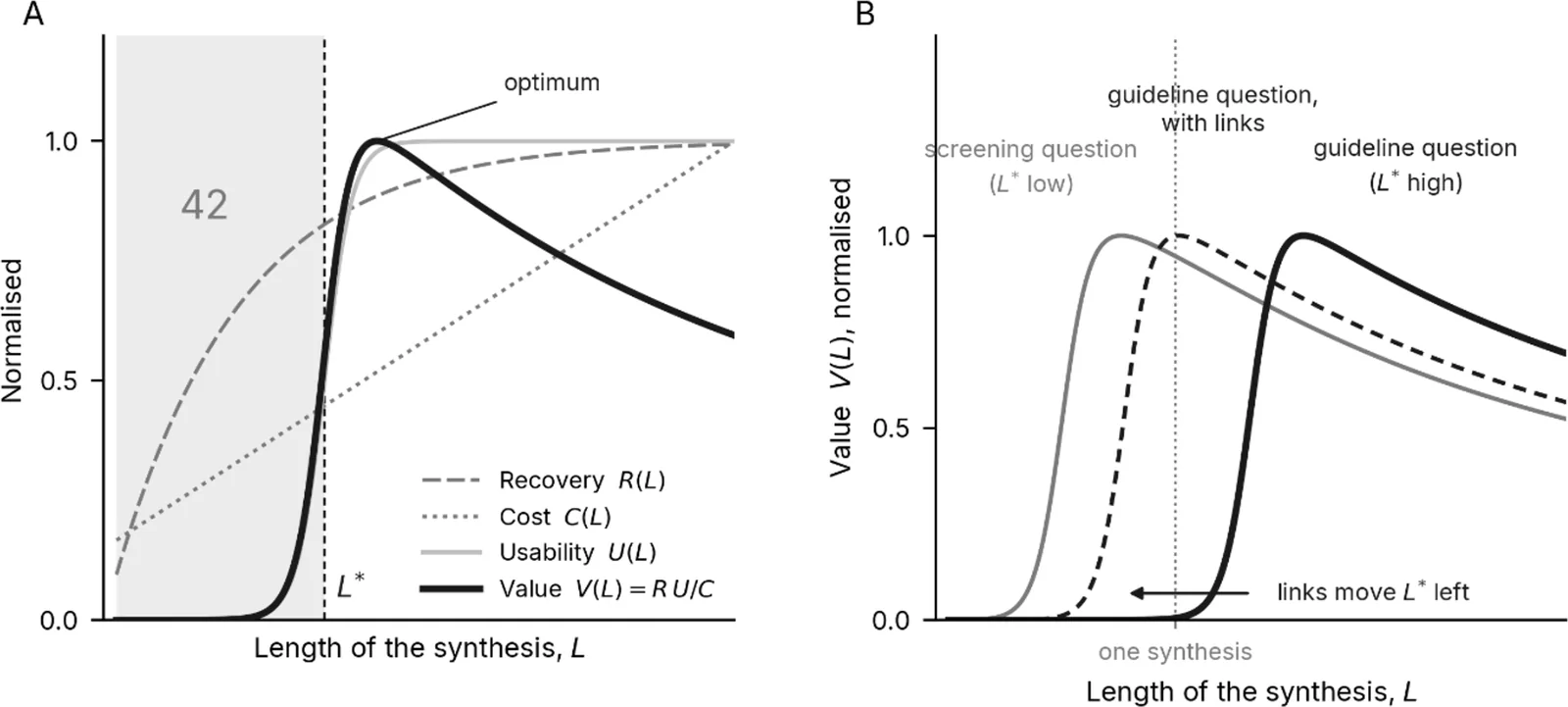
The value of a synthesis is not monotonic in its compression. (**A**) As the length L of a synthesis falls, recovery R(L) declines from saturation, while the reading cost C(L) falls steadily, so that value initially rises. This is the case for automated synthesis, and within this range it is correct. Usability U(L) holds near one until L crosses the threshold L*, below which the text no longer carries the conditions that make its claims valid, and V(L)=R(L)·U(L)/C(L) collapses. (**B**) The position of L* is set by the question and the reader, not by the text: the same synthesis sits above the cliff for a screening decision and below it for a clinical guideline. Links do not raise the curve; they displace context rather than destroying it, moving L* to the left and extending the range over which compression remains safe.

Two consequences follow. First, a system that does not know its reader cannot locate the cliff; it can only average over readers, which means being wrong about most of them. This is why "superhuman" is the wrong criterion. A system can be superhuman at retrieval but still fail at providing the right context of information for a particular reader and question. The second is a design claim, and the one we would like to put to anyone building these tools. Links to the sources do not raise the curve, i.e., they do not make a summary more valuable at any given length. They change how far it can be compressed before it becomes unusable, because the reader can recover the missing context through them (Figure 1B).

The strongest objection to our argument is not that the cliff does not exist but that we are comparing machine summaries against the wrong alternative. Critics of machine summaries – ourselves included – often evaluate them against what an expert would have written. However, more often the choice is between reading a compressed summary and reading nothing at all. Against that baseline, even a summary that overstates a finding may still be better than no summary.

And in a similar sense, in some tasks, machines have been shown to outperform experts. In a reader study involving ten physicians, summaries created by the best adapted models were judged equivalent to those written by medical experts in 45% of cases, and better in 36% (Van Veen et al., 2024). In systematic review screening, machine recall now matches or exceeds that of human reviewers, although at the cost of lower specificity [4]. "The machine loses the context, and the human keeps it" thus does not apply to summarisation in general. It holds for some tasks and not for others, and we have to say which.

The objection is valid, yet consistent with our argument. The curve already accounts for it: different tasks have different thresholds of compression (Figure 1B). Systematic review screening has a low L*: to decide whether a paper merits closer investigation, a reader needs to know the direction of its findings, not all the conditions under which those findings hold – heavy compression sits comfortably above the cliff. The same applies to the clinical texts in the physician study, which were written for readers with a relevant background needed to interpret the summaries. However, research findings are different. For scientific findings, their scope conditions are not merely a context that even an informed reader can fill in. They are part of the finding, and removing them may no longer report the original finding.

This is where the real danger lies, and where it has so far been overlooked. A summary created to screen papers carries no indication of the question it was compressed for. It may be saved in a reference manager, shared in a chat thread, and may eventually be used in a grant proposal, a guideline or a review. In those settings, the reader needs more context (i.e., the tolerable L* is far higher) and may not know the summary was never intended to provide. The summary outlives its purpose. Every summary that fails this way was initially a correct use of the tool – which is why better models will not fix the issue, and why an evaluation regime that scores summaries one at a time against their sources cannot see it at all.

We stress that this is merely a prediction and not an established finding: nobody has yet tracked a machine-made synthesis from the question it was built for to the document it ends up in. However, long before language models, a complete citation network built around a single claim has seen findings being restated without their scope conditions, hypotheses converted into fact by citation alone. The resulting distortions appeared in eight of nine funded grant applications, often used to justify the funding request (Greenberg, 2009). Machine synthesis adds volume and removes any mark of origin: across 2,046 empirical papers, 2.5% disclosed AI use at all, and none described how they verified the output (Ans et al., 2026).

Our own work sharpens this worry: people judge machine-written text – accurate or false – as more credible than human writing (Spitale et al., 2023), and models show similar patterns until the source is labelled, at which point their judgements skew (Germani and Spitale, 2025). Machines sound more accurate than humans do but are less reliable than they look once context enters. The remedy therefore is not better prose. Context is carried in the links, not the sentences: compression with recoverable context is the antidote; compression without it is 42.

A practical criterion for evaluating AI summaries is not fluency but recoverability of the claims. To make this criterion useful, we need measures that can be reported alongside a synthesis, without having to know the original question. We propose three:

**Coverage**: What proportion of the claims in a synthesis are linked to a source? A claim without a source cannot be traced to its source, regardless of its accuracy.

**Depth**: How much effort does it take to read the summary and retrieve the source links? A citation resolving to a forty-page report is linked in name only; one resolving to the warranting passage is attached in fact.

**Warrant**: Does the cited source, once reached, support the claim? This is where systems fail. Across 14 language models run as deep research agents (which search, retrieve and cite sources on their own), link validity exceeded 94% and topical relevance exceeded 80%, yet factual support for the citing sentence ranged between 39% and 77% (Onweller et al., 2026). More concerning still, as AI systems increased retrieval calls from two to 150, warrant fell by about 42%, even though link validity and topical relevance never dropped below 92%. In other words, retrieving more material did not produce more recoverable synthesis; it only yielded more citations that do not hold.

The advantage of these measures is that they do not require L* (the point at which compression becomes too great for a particular reader or task) to be standardised, because it is subjective to the reader. Coverage, depth and warrant, by contrast, describe properties of the synthesis and its links to the evidence. They can be measured and reported regardless of who will eventually read the summary or what they will use it for.

One of these measures already exists in similar forms. Other evaluations of deep-research systems measure warrant under different names (Onweller et al., 2026), and a survey of 134 papers identifies two warrant-type dimensions, attribution and citation, among the seven it uses to organise 300 evaluation metrics (Schreieder et al., 2026). But attribution is judged against retrieved sources, and citation against annotated question-answer-evidence triples. Both need an external evidence set, making them benchmark instruments rather than figures a tool can report.

The other two measures are largely absent. Existing evaluations ask whether supporting evidence exists somewhere in the retrieved material, not what proportion of a synthesis’s claims are individually linked to a source. Nor do they measure the effort a reader must expend to move from a claim to the specific passage that warrants it.

That is the change we are proposing: make recoverability a reported property of machine-generated synthesis. Journals already require its human equivalent. The ICMJE, for example, calls for "appropriate attribution of all quoted material, including full citations" (COPE, 2023; ICMJE, 2026). Reporting recoverability is based on the same principle: it makes the traceability of claims to evidence measurable, rather than leaving it as a matter of citation practice alone.

The ‘Ultimate Answer’ failed not because it was wrong, but because it arrived without context. As we hand more of our reading to machines, the danger is not that they will produce bad answers. It is that they generate fluent, concise and convincing answers detached from the questions and conditions that give them, and that these answers will travel far beyond the purposes for which they were created. The bottleneck is still us: our ability to ask the right questions, judge the evidence and turn information into understanding. And the tools worth building recognise this limitation and preserve our ability to recover what their summaries leave out.
